# Decreasing employees’ work stress by a participatory, organizational level work stress prevention approach: a multiple-case study in primary education

**DOI:** 10.1186/s12889-020-08698-2

**Published:** 2020-05-13

**Authors:** Maartje C. Bakhuys Roozeboom, Roosmarijn M. C. Schelvis, Irene L. D. Houtman, Noortje M. Wiezer, Paulien M. Bongers

**Affiliations:** 1 TNO (The Netherlands Organization for Applied Scientific Research), Leiden, Netherlands; 2 Erasmus University Rotterdam, Rotterdam, Netherlands; 3 Body@Work, Research Center Physical Activity, Work and Health, TNO-VU University Medical Center, Amsterdam, Netherlands

**Keywords:** Work stress, Job satisfaction, Primary education, Organizational level intervention

## Abstract

**Background:**

Work stress is an important problem among employees in education in the Netherlands. The present study aims to investigate the effects of a participatory organizational level work stress prevention approach to reduce (quantitative) job demands, increase resources (i.e. autonomy, supervisor and coworker support) and to reduce work stress and increase job satisfaction of employees in primary education.

**Methods:**

This study makes use of a multiple case study research design. The stress prevention approach is implemented at 5 primary schools and questionnaires were filled out by 119 employees of the 5 schools at baseline and 1 year later, measuring job demands, resources, work stress, job satisfaction and implementation factors.

**Results:**

Multilevel analyses showed a significant decrease in job demands and a significant increase in job satisfaction between baseline and follow up. In addition, employees that were more satisfied with the communication about the intervention showed more improvements in autonomy and job satisfaction. However, employees reporting an increased dialogue in work stress between employees and management showed a smaller decrease in job demands.

**Conclusion:**

The study shows a decrease in job demands and an increase in job satisfaction in the schools that implemented a stress prevention approach. Results of the study underline the importance of communication about the intervention as part of the implementation process, impacting the effectiveness of the intervention to improve autonomy and job satisfaction.

**Trial registration:**

ISRCTN registry, study ID: ISRCTN14697835, registration date: 11-10-2019 (retrospectively registered).

## Background

Among EU-workers, 25% consider their health to be at risk due to work stress [1], and this number is even higher for workers in education (42%) [2]. According to a survey that is representative for the Dutch workforce, one in five employees in education in the Netherlands actually suffers from work stress [3], i.e. they feel emotionally drained and exhausted especially at the end of the work day, and are tired when they get up again in the morning. In primary education, the target group of the present study, this would equal 32,165 of the 168,400 employed workers in 2017. In addition, at present there is a significant shortage of teachers in the Netherlands, especially in primary education and this problem is jeopardizing the quality of the Dutch educational system.

There is a lot of evidence that work stress causes major health problems, such as cardiovascular diseases [4–7], musculoskeletal disorders [8], and poor mental health [9]. Work stress is also found to increase sickness absence [10], decrease job satisfaction [11] and lower productivity [12]. Considering the severe consequences of work stress for employees and employers, it is important that organizations take measures to reduce these risks. The high prevalence of work stress in primary education, combined with the shortage of teachers in this sector, ask for effective interventions to reduce work stress and increase job satisfaction, to prevent teachers from leaving their profession.

In the last decades, a lot of research has focused on causes of work stress and several theoretical models have been developed (e.g. JDC(S)-model [13], the DISC-model [14] and JDR-model [15]). These models are all based on the balance principle: work stress as a result of excessive job demands combined with a shortage of available resources. Job demands are the physical, social or organizational aspects of the job that require effort [16]. Resources refer to aspects of the job that reduce job demands and the required efforts, help to achieve work goals and stimulate learning and development [17].

Job demands that have been found to correlate positively with teacher burnout are time pressure and work overload [18–21]. Resources that are found to be related to work stress in teachers are amongst others lack of autonomy [22, 23] and lack of supervisor support [20, 24].

According to the “hierarchy of controls” principle, interventions are presumed to be most (cost-) effective when work stress risks are managed at their source (i.e. primary prevention, aiming at job demands and resources) [25]. In addition, it is assumed that organizational interventions hold most potential for structural changes as opposed to individual interventions. These latter interventions may improve the well-being of individuals, but organizational interventions target the actual causes of stress, and may thus lead to substantial and sustainable improvements at both individual and organizational level. In practice, most interventions to prevent or reduce work stress in education focus on empowering individuals to deal with job demands. Different studies have shown only partial effects of these interventions on work stress [26–28]. Based on their review of organizational interventions aimed at reducing work stress in teachers, Naghieh et al. [29] conclude that organizational interventions lead to improvements in well-being of teachers, even though good quality effect evaluations of organizational interventions are scarce.

In the last decades, considerable efforts have been put into the consolidation of evidence concerning good practice interventions dealing with stress in the workplace. A large study on best practices of psychosocial risks (including work stress) management in Europe has resulted in a best practice framework for psychosocial risk management (PRIMA-EF) [30]. Based on interviews and focus group meetings, seven key features of work stress interventions have been identified. That is, interventions need to: 1) be theory and evidence-based; 2) follow a systematic, stepwise approach, including developing clear goals, tasks and intervention-planning; 3) apply a proper risk assessment, identify risk factors and vulnerable groups; 4) be tailored to the organizational context (e.g. sector, size, culture), and be adaptable and flexible; 5) be accessible and user friendly; 6) be targeted at the individual as well as the organization, and 7) develop (management and leadership) capacities and skills. Several interventions that include these features have been tested [31–34]. In most cases, the intervention consists of several steps that can be summarized as: a preparation phase, a risk assessment phase, an action planning phase, an implementation phase, and an evaluation phase. The first three steps of the intervention, the preparation phase, risk assessment phase and the action planning phase, result in a tailored action plan that targets organization specific stressors or hindrances. Implementation of this action plan is – in line with the Job Demands Resources model – hypothesized to reduce job demands and increase resources, which will in turn decrease levels of work stress and increase job satisfaction [16, 17, 35].

However, the implementation of these interventions is complex [36] and the success of such interventions depends on many factors [37, 38]. Several implementation factors that appear to be important for the success of the intervention are employee participation, communication, and dialogue [39]. Implementation factors are not only considered to be crucial for successful implementation, but these factors in themselves can be considered as active ingredients of the intervention since they provide resources for employees. The process evaluation model of Nielsen & Randall [40] identifies participation of employees during the implementation process as an important driver of change. Employee participation is important because employees have expert knowledge of the workplace and work processes, and by involving them in managing psychosocial risks this knowledge is accessible [31, 34]. In addition, participation of employees in the intervention, provides opportunities for employees to control their working conditions and “worker control” is an important determinant of employee wellbeing [13]. Furthermore, involving employees in identifying stressors and finding solutions will increase employees’ readiness for change and ensure commitment for the implementation of the measures. For these reasons, the participatory approach has been broadly advocated as an effective strategy in organizational interventions to improve occupational health [34]. Another important implementation factor mentioned in previous research is clear and transparent communication [39, 41–43]. Communication about and throughout the process is very important to get and keep employees informed and involved. Communication about the intervention and the intervention process contributes to employees’ understanding of the intentions behind the interventions, increasing employee participation in and commitment to the intervention [44]. In their model of process evaluation, Nielsen & Randall [40] consider communication to be a crucial aspect of the implementation strategy. In addition, Nielsen & Randall [40] stress the importance of the perceptions and appraisals of individuals in the organization towards the intervention, since these so-called mental models determine how individuals behave and react to the intervention. Different individuals in the organization (e.g. employees, supervisors, management) can have different and conflicting agendas. Aust et al. [45] showed that differences in stakeholder views may hinder successful implementation, stressing the importance of shared mental models of individuals in the organization towards the intervention. The dialogue on stress among employees and between employees and management can contribute to shared mental models and facilitate the implementation. Other researchers also stress the importance of the dialogue between management and employees as a driver for organizational improvement regarding the work environment and employee health [39, 41, 43, 46].

Not only is the implementation of an organizational level work stress intervention difficult, the evaluation of intervention effects is challenging as well. In applied research, the research design has been a topic of discussion for years. Traditional research designs in the psychology and health domain are experimental designs and randomized controlled trials (RCT), usually involving a pre- and posttest, an experimental (or intervention) and a control group and random assignment of respondents or research units to the experimental (or intervention) and control group. These research designs are by many considered as the golden standard. However, in applied organizational research, these research designs are often not feasible since (quasi-)experimental designs with a control and experimental group are often difficult to establish and the organizational context is often complex and therefore hard to control, making extrapolation of the results to other organizations and individuals difficult [47–50]. Randall, Griffith and Cox [48] propose an alternative research design to cope with these problems, that better fits the organizational context, by using the results of the process evaluation of the implementation (measuring e.g. participant’s participation and intervention exposure as a proxy of the level of implementation) in the effect evaluation. Huijs et al. [51] followed a similar approach by using data obtained in a process evaluation of participants’ experiences and exposure to the intervention and investigated whether changes in the outcome measure between baseline and follow-up were related to the level of intervention exposure. Following this approach provides the possibility to account for the complex and often uncontrollable organizational setting.

### Work stress prevention approach

For the present study, a work stress prevention guideline for intervention facilitators (e.g. internal HR-advisor or external consultant) was developed, based on the above described existing knowledge. The guideline is designed as an interactive pdf document, in order to tailor information based on the facilitator’s prior knowledge of the topic. The guideline provides a detailed description of a participative, five-step approach to prevent work stress (the work stress prevention approach), including per step what to do, how to do it, when to do it and with whom. And since the implementation factors described earlier are considered very important for the success of the intervention, the guideline provides information and inspiration to enhance employee participation, to provide employees with clear communication during the intervention and to improve the dialogue on work stress within the organization. Following the work stress prevention approach results in a tailored action plan for each school, that addresses school specific risk factors (in terms of job demands and resources).

The work stress prevention approach consists of five successive steps aiming to facilitate the formulation, implementation and evaluation of specific work stress measures. These steps are: 1) preparation, 2) risk assessment, 3) action planning, 4) implementation, and 5) evaluation. In all the five schools that participated in this study, the implementation process of the approach is facilitated and coordinated by the same intervention facilitator. The intervention facilitator is experienced in change- and project management and received three two-hour training sessions on the work stress prevention approach by the researchers. In this training the approach is explained in detail, and special attention is paid to the important implementation factors: employee participation, communication and the dialogue on stress. The facilitator follows the protocol as described in the work stress prevention guideline.

Step 1 entails the preparation phase. In this phase a working group is formed in each school consisting of the director, 1–3 workers with an interest in the topic of work stress and the intervention facilitator. The working group is responsible for facilitating steps 1–5 to be followed in their own school, involving and informing employees and monitoring the implementation process. The working group decides upon a suitable communication strategy to keep employees informed during the intervention process (e.g. weekly newsletters, posters in the staff room, presentation at personnel meetings). A kick-off meeting is organized and the project is announced by the working group to all employees. Tasks of the working group are performed within working hours.

In step 2 - the risk assessment phase - causes of work stress are examined. For this purpose a questionnaire is administered by the researchers with amongst others questions on determinants (job demands and resources) and on outcomes (work stress and job satisfaction) (see paragraph on measures). Results of the baseline questionnaire are benchmarked against data representative for the entire Dutch primary education sector, based on the Netherlands Working Condition Survey [3] in order to prioritize the factors causing work stress. In addition, a participatory focus group session is organized with all personnel to present and discuss the results of the questionnaire, to check whether the priorities based on the numbers relate to their experience of the causes of stress and to identify additional causes of stress (if any) in their school.

In step 3, the action planning phase, work stress measures are jointly developed. In a brainstorm session with all personnel an extensive list with all possible solutions based on expert knowledge of the participants about their working environment was formed (divergent technique). Next, a selection of the 5–10 most appropriate and feasible work stress measures is made (convergent technique). Based on this selection a detailed action plan is developed by the working group under supervision of the facilitator.

Step 4 - the implementation phase - entails the implementation of the measures as described in the action plan resulting from step 3. The working group implements the measures according to the action plan and regularly discusses progress and communicates about the process to the employees.

In step 5 - the evaluation phase - the effects of the work stress prevention approach and the implementation process are investigated. A follow up questionnaire, the same as the baseline questionnaire, is administered, and 4 interviews are conducted per school by the researchers. Results from the questionnaire and interviews are discussed with the working group by the facilitator to evaluate the success of the measures and to decide upon next steps. Results of the questionnaire and interviews are also shared with all personnel.

The current study aims to explore the effect of the work stress prevention approach on (quantitative) job demands and resources (autonomy, supervisor and coworker support) and on work stress and job satisfaction. The current study follows a similar approach as Huijs et al. (2019) by investigating the effects of the intervention in relation to the implementation success, as measured by the level of employee participation, communication and dialogue on stress.

The study examines the effects of the work stress prevention approach as a whole, rather than the effects of specific measures as described in the school specific action plans (result of Step 3).

Each school developed or selected their own measures, and as a result of the variation in contexts and priorities there is also a variation of different kinds of measures, making it difficult to examine the effects of separate measures. The authors believe that the effects of the stress prevention approach is related to the approach as a whole. The fact that the measures as determined in the action planning phase are tailored to school specific problems is considered more important than the exact content of the measures.

**Fig. 1:**
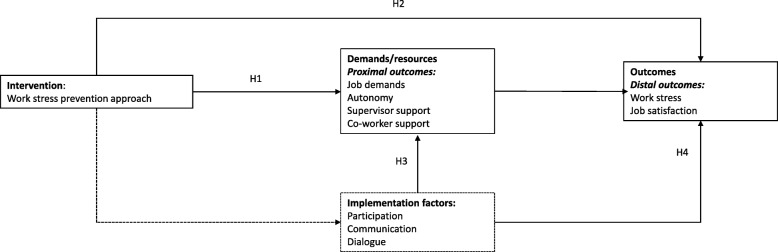
Schematic overview of hypotheses

Based on the above, the following hypotheses were formulated (see Fig. 1):

- Hypothesis 1 (H1): The level of job demands will decrease and resources (autonomy, supervisor and coworkers support) will increase between baseline and follow-up (proximal outcomes)
- Hypothesis 2 (H2): Work stress will decrease and job satisfaction will increase between baseline and follow-up (distal outcomes).
- Hypothesis 3 (H3): The implementation factors (participation, communication and dialogue on stress) will positively affect the decrease in job demands and the increase in resources (proximal outcomes) between baseline and follow-up.
- Hypothesis 4 (H4): The implementation factors (participation, communication and dialogue on stress) will positively affect the decrease in work stress and the increase in job satisfaction (distal outcomes) between baseline and follow-up.

## Methods

### Study population

The study population consisted of teaching (i.e. teachers) and non-teaching staff (i.e. managers, support staff) from five schools in primary education (*N* = 119). Schools were recruited via the network of the primary education labour market platform (Arbeidsmarktplatform Primair Onderwijs) by placing an advertisement in a sector specific magazine. Five schools applied for participation. Reasons for participation were amongst others signals of work stress reported by employees. The schools were geographically spread throughout the Netherlands. The schools differed in size, and included small, medium and large schools (teaching and non-teaching staff at baseline: school A: *N* = 15, school B: *N* = 61, school C: *N* = 45, school D: *N* = 37 and school E: *N* = 41). The study did not require ethical approval, since the study did not fall under the Medical Research Involving Human Subjects Act (WMO) [52, 53].

### Data collection

A digital questionnaire was sent out by email to all personnel of the five primary schools as part of step 2 ‘risk analysis’ (baseline) and step 5 ‘evaluation’ (follow-up) of the work stress prevention approach. The baseline questionnaire was sent out in March 2016. The follow up questionnaire was sent out 12 months after the baseline questionnaire. Data on proximal outcomes (job demands and resources) and distal outcomes (job satisfaction and work stress) were collected by means of the baseline and follow up questionnaires. Data on implementation factors were collected by means of the follow up questionnaire.

### Measures

#### Job demands and resources (proximal outcomes)

Job demands and resources are measured using a proxy of subscales of the Dutch version of the Job Content Questionnaire (JCQ [54]): quantitative job demands (4 items; α = .84) and resources: autonomy (3 items, α = .67), supervisor support (4 items; α = .77) and co-worker support (4 items: α = .73). Response scales range from 1 = *strongly disagree* to 4 = *strongly agree*.

#### Outcome variables (distal outcomes)

Work stress was measured with a shortened version the Utrecht Burnout Scale (UBOS) [55], a slightly adjusted Dutch version of the Maslach Burnout Inventory-General Survey (MBI-GS) [56]. The questionnaire consists of 5-items including the key dimension of burnout: emotional exhaustion (feeling drained by one’s work). Response scales range from 0 = *never* to 6 = *every day* (α = .84). Studies have shown that the MBI-GS and its subscales are excellently reliable and valid [57, 58].

Job satisfaction can be viewed as a general and one-dimensional construct, resulting from positive and negative work experiences [59]. It was measured with one item: *“I am satisfied with my present job”*. This item was rated on a 5-point Likert scale, response scales range from 1 = *strongly disagree* to 5 *= strongly agree*.

#### Implementation factors

The follow up questionnaire contained the following items on the implementation that are used in the analyses to indicate the implementation success: the level of employee participation, communication and dialogue on stress. Employee participation was assessed by a single item*: "Could you rate your involvement with the intervention program on a scale from 1 (=poor) to 10 (=excellent)?"* Communication was measured by a single exploratory item: "*Could you rate your satisfaction with the communication about the intervention program on a scale from 1 (=poor) to 10 (=excellent)?"* Dialogue on stress was measures by three separate items. Respondents were asked to indicate on a 5-point Likert scale (response scales range from 1 (strongly disagree) to 5 (strongly agree)) “to what extent did you notice any changes regarding the following areas?”: “*Work stress is discussed more often among employees*” (dialogue between employees); “*Work stress is discussed more often between employees and management*” (dialogue with management); “*There is more attention for the issue of work stress throughout the school*” (attention for work stress).

### Data analyses

Analyses were performed on the data of the five primary schools combined. To adjust for clustering of persons in schools, multilevel analyses were performed using IBM Statistics SPSS version 25.0. Multilevel modelling can be used to analyze data that contain an inherent hierarchical structure. The data from the current study contain two levels: the first level of the data contains the individual scores of the participants on the proximal and distal outcomes at baseline and follow-up (within-subjects level) and the second level of the data contains the schools in which the individual participants are nested (between schools level). To start, the variables have been prepared for analyses. For all the variables a new ‘centered’ variable was calculated, by subtracting its mean from each individual score, to make the interpretation of the output of the analyses more straightforward. For each outcome a random intercept was added to the model to adjust for differences between the schools in the way the proximal and distal outcomes changed over time.

To test hypotheses 1 and 2, difference scores (between baseline and follow-up) were calculated for each outcome. Univariate analyses were carried out with the difference scores of each of the proximal (job demands, resources) and distal outcomes (work stress and job satisfaction) as dependent variable; the centered score of the outcome at baseline as the independent variable and the intercept to indicate the average change in the outcome between baseline and follow up. In the analysis covariates were added based on differences between schools regarding the baseline measurement of general characteristics. These analyses test the difference between baseline and follow up for each of the proximal and distal outcomes corrected for age and the outcome at baseline.

In addition, the analyses of the previous step were repeated including the centered implementation factors as covariates. These analyses test hypotheses 3 and 4, and show whether a difference between baseline and follow up in the proximal and distal outcomes (job demands, resources, work stress and job satisfaction) was moderated by the implementation factors (participation, communication and dialogue) controlling for covariates (differences between schools on the baseline measure of general characteristics) and the outcome at baseline. To obtain the amount of variance explained by the differences between the schools, the intraclass correlation coefficient (ICC) was calculated for each analysis. For all hypotheses a *p*-value of < 0.05 was indicated as statistically significant.

### Qualitative analyses

In addition to the quantitative data that were collected to explore the effects of the intervention and test the hypotheses, also qualitative data were collected to explore the implementation process in more detail. Qualitative data on the implementation process were collected during Step 5 by four semi-structured interviews in each primary school on the experience of various employees with different roles during the implementation of the approach. These interviews were conducted by the researchers. In each school interviews were held with the director, a working group member, a randomly selected worker not taking part in the working group, and the intervention facilitator who accommodated all five schools. The interviews were conducted according to a semi-structured interview protocol, either by telephone (*n* = 15) or face to face (*n* = 5), and lasted between 30 and 60 min. Minutes were made during the interview by a research assistant. The interview transcripts were coded according to different topics that were determined beforehand: experiences with the five phases of the work stress prevention approach and the actions within each phase (questionnaire, focus group meeting, brainstorm session, conducting action plan, progress meetings, role of intervention facilitator, role of working group, participation of employees), drivers and barriers for implementation of the work stress prevention approach and strengths and weaknesses of the work stress prevention approach (the semi-structured interview protocol is added as supplementary file).

## Results

**Fig. 2:**
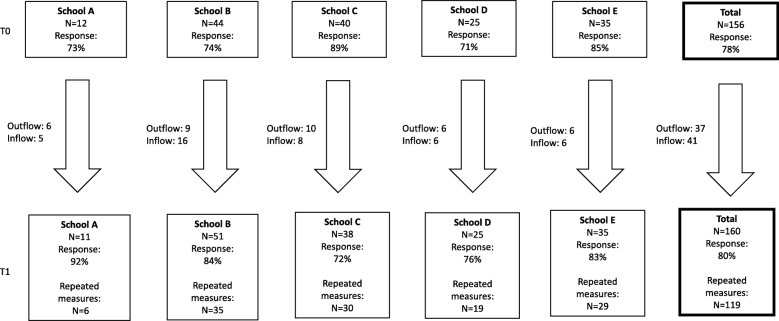
Flow-chart of response rates for the five primary schools

Figure 2 shows the participant flow and response rates of the baseline and follow up. At baseline, the response rate was 78% (of all eligible workers), and at follow up the response rate was 80% (of all eligible workers). In total 119 respondents completed both baseline and follow up and were included in the analyses since this is the group for which repeated measure analyses could be performed.

Table 1 shows general personal characteristics of the study population. There are some statistically significant differences between schools in relation to several of these characteristics, particularly regarding age. For this reason, age was added as a covariate in the analyses. There are no statistically significant differences on the baseline proximal and distal outcome measures between the schools.

**Table 1:** General characteristics of study population

		School				
	Total	School A	School B	School C	School D	School E
N:	119	6	35	30	19	29
% of total sample:	100%	5%	29%	25%	16%	24%
Gender [N = 119]						
Male	9.2%	16.7%	14.3%	3.3%	5.3%	10.3%
Female	90.8%	83.3%	85.7%	96.7%	94.7%	89.7%
Age (in years) [N = 119]						
20–30	15.1%	0%	11.4%	10.0%	36.8%▲	13.8%
30–40	31.1%	16.7%	42.9%	40.0%	15.8%	20.7%
40–50	18.5%	16.7%	11.4%	20.0%	10.5%	31.0%▲
50–60	29.4%	16.7%	28.6%	26.7%	36.8%	31.0%
+ 60	5.9%	50.0%▲	5.7%	3.3%	0%	3.4%
Position [N = 119]						
Teacher	85.7%	100%	88.6%	76.7%	84.2%	89.7%
Staff	10.1%	0%	11.4%	16.7%	5.3%	6.9%
Management	4.2%	0%	0%	6.7%	10.5%	3.4%
Job demands (range 1–4, 4 items)[N = 119]						
Mean	2.74	2.75	2.82	2.69	2.59	2.80
Standard deviation	0.60	0.84	0.63	0.47	0.67	0.60
Autonomy (range 1–3, 3 items)[N = 119]						
Mean	2,34	2,17	2.26	2.46	2.40	2.32
Standard deviation	0.47	0.75	0.52	0.40	0.42	0.44
Supervisor support (range 1–5, 4 items)						
Mean	2.99	3.08	2.90	2.83	3.20	3.09
Standard deviation	0.63	0.34	0.76	0.57	0.52	0.57
Co-worker support (range 1–5, 4 items)						
Mean	3.37	3.58	3.36	3.43	3.28	3.36
Standard deviation	0.45	0.34	0.49	0.38	0.52	0.46
Work stress (range 1–7, 5 items)						
Mean	2.68	3.47	2.81	2.41	2.60	2.70
Standard deviation	1.17	1.52	1.30	1.21	0.89	1.04
Job satisfaction (range 1–5, 1 item)						
Mean	3.79	3.33	3.69	3.90	3.95	3.79
Standard deviation	0.78	1.21	0.96	0.71	0.40	0.68

Percentages are column percentages and are tested with the Pearson χ^2^-test (horizontal comparisons). The contrast is subgroup vs ‘rest’ (weighted deviation contrast). ▲ and ▼: *p* < 0.05, significant high (low) percentages (two-tailed), and Cohen’s d is at least 0.20

### Quantitative analyses

Table 2 shows the results of the analyses performed to test H1 and H2. Even though in the analyses we corrected for clustering effects of school by means of a multilevel approach, for job demands, co-worker support, work stress and job satisfaction no differences were found between the schools (Table 2). Results show a statistically significant decrease in job demands and increase in job satisfaction from baseline to follow up, partly confirming H1 and H2. All other proximal and distal outcomes appear to have changed between baseline and follow up in a favorable direction, although these results are not statistically significant (for work stress the effect is marginally significant, *p* < .10).

**Table 2:** Effects of the work stress prevention approach on the difference scores of the proximal and distal outcomes (H1 and H2)

	H1				H2	
	Job demands B (95% CI)	Autonomy B (95% CI)	Supervisor support B (95% CI)	Coworker support B (95% CI)	Work stress B (95% CI)	Job satisfaction B (95% CI)
Intercept	−.10* (−.20–.01)	.05 (−.11–.21)	.15 (−.11–.41)	.03 (−.03–.11)	−.15 (−.32–.03)	.12* (.02–.21)
Baseline of outcome measure	−.70* (−.86 - -.54)	−.47* (−.63--.31)	−.58* (−.73--,43)	−.52* (−.67--.37)	−.27* (−.42--.12)	−.43* (−.55--.31)
Age	−.08 (−.16–.00)	−.03 (−.09–.04)	.08 (−.01–.16)	.01 (−.05–.07)	−.18* (−.33--.03)	.04 (−.04–.12)
ICC	~.00	.03	.07	~.00	~.00	~.00

*p < 0.05

Table 3 shows the results of the analyses performed to test H3 and H4 indicating an effect of the implementation factors on the change in proximal and distal outcomes between baseline and follow up. Results show that the implementation factor communication affects the differences between baseline and follow up on job satisfaction and autonomy. Respondents who were more satisfied with the communication about the work stress prevention approach, showed a larger increase in job satisfaction and autonomy between baseline and follow up, than respondents who were less satisfied with the communication.

**Table 3:** Results from multivariate mixed model multilevel analyses on H3 and H4

	H3				H4	
	Job demands B (95% CI)	Autonomy B (95% CI)	Supervisor support B (95% CI)	Coworker support B (95% CI)	Work stress B (95% CI)	Job satisfaction B (95% CI)
Intercept	−10* (−.19 - -.01)	.04 (−.16–.24)	−15 (−.10–.40)	.04 (−.03–.11)	−.15 (−.32–.03)	.11 (−.14–.36)
Baseline of outcome measure	−.70* (−.86 - -.53)	−.47* (−.62 - -.32)	−.64* (−.80 - -.48)	−.58* (−.74 - -.42)	−.32* (−.47 - -.15)	−.44* (−.56 - -.32)
Employee involvement	−.03 (−.11–.06)	−.02 (−.08–.05)	.05 (−.03–.13)	−.01 (−.07–.05)	−.01–.17–.15)	−.08 (−.16–.01)
Communication	−.00 (−.10–.10)	.08* (.01–.16)	.01 (−.09–.11)	.05 (−.02–.13)	−.06 (−.25–.13)	.13* (.03–.23)
Dialogue among colleagues	.03 (−.09–.16)	.06 (−.03–.15)	−.05 (−.17–.07)	−.00 (−.09–.09)	.03 (−.20–.25)	.10 (−.02–.22)
Dialogue with management	.15* (.01–.29)	−.02 (−.13–.09)	.09 (−.05–.23)	.00 (−.10–.11)	.08 (−.19–.34)	−.06 (−.19–.08)
Attention for work stress	−.09 (−.23–.05)	.06 (−.05–.16)	−.02 (−.12–.16)	.05 (−.05–.15)	−.14 (−.40–.12)	.03 (−.11–.16)
Age	−.07 (−.15–.02)	−.03(−.09–.03)	.08* (.00–.16)	.00 (−.06–.06)	−.16* (−.31 - -.01)	.04 (−.04–.12)
ICC	~.00	.08	.09	~.00	~.00	.01

*p < 0.05

Finally, results show that the ‘dialogue with employer’ affects the differences between baseline and follow up on job demands. The direction of this effect was in contrast to the hypothesis and indicates that respondents who did report an increased dialogue between employees and their employer regarding work stress, showed a smaller decrease in job demands between baseline and follow up, compared to respondents who reported no increased dialogue between employees and their employer regarding work stress.

To summarize, the results show a statistically significant decrease in job demands and an overall increase in job satisfaction between baseline and follow up, partly confirming H1 and H2. And satisfactory communication about the work stress prevention approach is related to an increase in job satisfaction and autonomy between baseline and follow up. In contrast to our expectations, results show that an increased dialogue between employees and the management is related to a smaller decrease in job demands between baseline and follow up. H3 and H4 are partly confirmed.

### Qualitative analyses

#### Preparation phase

At all schools, a working group was installed according to protocol, with the director, 1–3 workers, and the intervention facilitator.

#### Risk assessment phase

The response on the baseline questionnaire was quite high (response rates ranges 71–89%). In the interviews, respondents mentioned that they appreciated that the questionnaire provided ‘objective’ data on this sensitive topic of work stress, which provided a good starting point for discussion in the focus group sessions. In the focus group sessions, the participants valued the fact that they could provide input regarding the risk assessment, and that their view on work stress risks was taken into account. The risk factors for work stress at the schools were relatively similar, although there were some differences in relation to unwanted behavior from external persons (e.g. parents) which was particularly a problem for two of the five schools (Table 4).

**Table 4:** Action plans of the 5 schools

	School A	School B	School C	School D	School E
**Causes of work stress**					
High job demands	X			X	X
High administrative load	X	X	X	X	X
High time pressure	X	X	X	X	X
Work home interference		X			
Unwanted behavior from external persons		X		X	
High burden of non teaching tasks		X	X	X	X
Lack of support in non teaching tasks			X		X
Lack of support in administrative tasks	X	X		X	
Level differences of students		X			
Combination groups (students from two different school years combined in one group)			X		
Inefficient meetings	X				X
Difficult student population				X	
Lack of management support					X
Working overtime					X
**Categories of measures**					
Year Planning/ group plans/ work tasks	**Year planning:** Making framework for year planning Describing year tasks for each group Yearly evaluation and update of year planning **Work tasks:** Make list of current assignments, evaluate and update list Feedback training for teachers New colleagues are assigned to a mentor (the mentor receives time for mentor tasks) Teachers from year groups make a list of all assignments, log in codes etc	**Year planning:** Make a plan to reduce peak load Keep space in year planning to deal with peak load Cancel one meeting with parents Individual meetings between teachers and direction to discuss year planning	**Year planning:** Agreements with team about deadline of year planning, making adjustments throughout the year, be critical about what to include in year planning, arrange day for part-timers **Group plans:** Make, evaluate and adjust year plans, make sure teachers know how to use them **Work tasks:** Prioritizing work tasks and reduce unnecessary work tasks Divide work tasks based on teachers’ skills and preferences Make proportionate distribution of work tasks Keep space for unforeseen tasks Log all agreements in document	**Year planning:** Compare year planning of different school years and align them	**Year planning:** Making format for year planning per group Uniformity in conducting year planning Evaluation and update format **Group plans:** 4 moments per year for conducting and evaluating group plans
Administrative tasks	**Administrative tasks** Agreements on checking students work Investigating possibilities for digital tests to reduce task of checking students work Meetings with teachers to reduce double administration Improving data storage Make overview of administrators	**Administrative tasks** Evaluate and improve report form on student development (only reporting the necessary) Make appointments about informing colleagues that were absent at meeting Make format for scenario and adjust existing scenario’s based on new format and collect scenario’s	**Administrative tasks:** Make, evaluate and bundle agreements about how to work with Parnassys (digital report system) Make agreements on checking students work and discuss with teachers Outsource administrative tasks to administration officer	**Administrative tasks:** Adjusting group overview (containing only information that is not logged elsewhere) Change group plans, students are monitored in a different way Reducing 3 documents on group plan ‘behaviour’ into 1. Reducing checking students work Making clear the administrative tasks per function	**Administrative tasks:** Teachers plan 1 h per week to work on group plans
ICT	**Improvements in ICT:** Letter to the direction Meeting with ICT professional Making and implementing project plan to solve ICT issues				
Study days/ meetings	**Alternative program study days:** Explanation of the purpose of each educational activity Evaluation of study days and improving program based on evaluation Evaluation of other educational activities Discussion about planning parents meetings (afternoon of evening)	**Effective meetings:** Make agreements about effective meetings	**Effective meetings:** Make agreements on number of targets, number of meetings, content of meetings, involve teachers in meetings, making meetings more motivative		**Alternative program study days:** Study days will have practical and substantive component At least 45 min are available for practical issues **Effective meetings:** Conduct action list and use it at team meetings Team meetings will have practical component Education will be prepared in unit meeting that is already planned
Fit between education and student population		**Dealing with level differences of students:** Continuing existing program Execute and evaluate pilot ‘calculation’	**Adjusting education to student population:** Long-term program (more practical classes, more continuency regarding substitution, etc)	**Adjusting education to student population:** Improving tailoring to students needs Plan energizers in between lessons to motivate children.	
Unwanted behavior/ parents involvement	**Measures for unwanted behavior from parents:** Raising awareness (information in school guide, newsletter and incidental personal talks) Conducting protocol for unwanted behavior and discuss it in meeting with teachers Preparing hand-out with behavioural rules for teachers, students and parents	**Measures for unwanted behavior:** Recap of skills that are obtained in earlier training Inform teachers on protocol in case of unwanted behavior Publication of protocol in school guide and news letter **Improvement of parents involvement:** Conducting and using parent forms Inviting parents to theme meetings	**Proactive attitude in regards to parents:** Issues regarding unwanted behavior from parents are inventoried, teachers can get assistance when wanted Agreements are made about contact with parents **Improvement of parents involvement**: Organising two parent meetings	**Measures for unwanted behavior:** Protocol to deal with escalation Communicating protocol to parents	
Culture		**Empowerment teachers** Teachers can choose from educational activities in relation to time management and prioritization If teachers experience a problem, they are invited to propose a solution	**Investing in positive work climate:** Improve feedback culture among teachers Closing school day with students in positive manner	**Investing in positive work climate:** Teachers discuss school conduct rules with students Students are approached positively and are motivated to commit to school rules **Empowerment teachers:** Feedback training (to improve feedback culture among colleagues) Time management training	**Investing in positive work climate:** Make project plan to develop, implement culture card that presents the ideal work culture **Increasing employee commitment:** Teachers are asked to submit creative idea for division of work tasks During study day, tasks are divided by means of task market
Leadership	**Leadership:** Communication on important decisions from direction to teachers Teambuilders assist at meetings Teamleaders make workplace rounds between 8.00–8.15 o clock				**Leadership:** Direction is visible at workplace – workplace rounds Development of employees is discussed in development meeting with direction

#### Action planning phase

At all schools, almost all personnel participated in the brainstorm session. At two schools the brainstorm session was combined with the focus group session. Participants valued the possibility to give their input regarding the measures which were considered needed. According to the intervention facilitator, the commitment of participants of the focus group meetings and the brainstorm sessions was high. Based on the results of the brainstorm session, the working groups developed an action plan. The schools differed in relation to the measures identified as well as to the persons who were made responsible for the implementation of the measures (Table 4). At some schools the implementation of the action plan was delegated among several persons, at other schools only one or two persons were made responsible.

#### Implementation phase

The implementation phase was considered by the intervention facilitator as the most difficult phase. Different progress meetings were planned with the working groups to discuss progress, and to discuss drivers and barriers of the implementation. The most often mentioned barrier for the implementation of the action plan was lack of time and lack of priority. The progress meetings and regular talks between the working group and the intervention facilitator stimulated the working group to give priority to the implementation of the action plan. The working group members and the intervention facilitator mentioned that it was challenging to keep all personnel informed and involved. Several communication channels were used to inform personnel (e.g. newsletters, meetings, blogs, flip-overs in staff room).

#### Evaluation phase

Comparable to the baseline questionnaire the response of the follow-up questionnaire was high (response rate ranged from 72 to 92% per school). In the interviews, the participants were asked whether they had noticed effects from the work stress prevention approach. The results were somewhat inconclusive. Participants valued some of the concrete measures (e.g. more efficiency in administration, meetings and checking students results). But some argued that important determinants of work stress are out of the reach from the primary schools (e.g. some of the administrative tasks are obliged). Participants explicitly mentioned the value of the participative approach and they mentioned that the dialogue on stress within the school helped to raise awareness and making stress prevention a shared responsibility.

## Discussion

The aim of the present study was to explore the effect of the work stress prevention approach on job demands, resources, work stress and job satisfaction in five primary schools and to investigate whether and how implementation factors were related to these effects. The study investigated the effects of the approach as a whole, rather than the school specific measures as described in the action plans of the schools. Despite the fact that the schools conducted different action plans, the analyses showed that differences between schools in relation to the effects of the work stress prevention approach were small or absent.

Quantitative analyses were performed to test whether there was a positive change between baseline and follow-up in job demands and resources (H1) and whether there was a decrease in the level of work stress and an increase in the level of job satisfaction (H2) after the intervention. Results of the analyses showed no significant changes for resources (autonomy, supervisor and co-worker support) and work stress, but there was a statistically significant decrease in job demands and an increase of the level of job satisfaction, partly confirming H1 and H2. From the literature it is known that job satisfaction is an important predictor of company performance [60], and high job satisfaction decreases turnover intention [61]. This finding could form an argument for making a business case for stress management, encouraging employers to take action.

Although the study found a decrease in job demands, and in job satisfaction no significant changes in resources and work stress were found between baseline and follow up. A possible explanation is that the follow up questionnaire was conducted too early in time to be able to show any significant changes in these indicators since the implementation process may have been slow and actual changes might only have just started. In addition, the implementation of the interventions followed the same steps on each of the five schools, but the timing of the steps was not exactly the same. According to De Lange et al. [62] the time interval between baseline and follow up is ideally 1 year, and a similar time interval was applied in the present study. However, the cyclical character of the work stress prevention approach makes it difficult to determine a good timing for the follow up, since ideally the approach does not end, but will be adopted as part of the policy cycle within the organization. At the time of the follow up questionnaire all schools were still implementing measures from their action plans, but some of the schools had already implemented more measures than others. Furthermore, the effects of some of the measures could be assumed to manifest themselves earlier than the effects of other measures. For example, reducing unnecessary work tasks may have had an immediate effect on job demands, but increasing social support and autonomy may take more time.

Looking at the difference between the resources and outcomes at baseline and follow up, all changed in a favorable direction, however, the changes were not statistically significant with the exception of -as indicated before- job demands and job satisfaction (for work stress the effect was marginally significant, *p* < 0.1). It is possible that, if there had been more time between baseline and follow up, more measures from the action plans could have been implemented, and possibly more effects of the measures on resources and work stress would have been found. On the other hand, it is also possible that by postponing the follow up, some of the effects may already have faded away.

To attribute changes between baseline and follow up to the intervention, the changes on the proximal and distal outcomes between baseline and follow up were related to the implementation factors: employee participation, communication and the dialogue on stress. The assumption was that, when employees participated in the intervention, were satisfied about the communication, and the dialogue on work stress had increased during the intervention, this would form a proxy of implementation success, and the intervention would be more likely to result in positive effects on job demands and resources (H3) and on work stress and job satisfaction (H4).

In line with these hypotheses, results of the quantitative analyses suggest that employees who are more satisfied with the communication about the intervention, appeared to have benefited more from the intervention. Results show that the level of satisfaction with the communication over the intervention did affect favorable changes between baseline and follow up in autonomy and job satisfaction.

The level of participation of employees in the intervention did not appear to affect changes on job demands, resources and outcomes. Regarding the dialogue on stress, the results were somewhat inconclusive. Although participants explicitly mentioned the dialogue on stress within the school as a key feature of the work stress prevention approach, the results of the analyses show that in fact the level in which the intervention increased the dialogue on work stress between employees and management, was related to less of an increase in job demands between baseline and follow up. Respondents who reported an increase regarding the dialogue between employees and management on work stress showed less of a decrease in job demands. A possible explanation is that the dialogue between the employees and management may have led to extra tasks, at least at short term. Discussing work stress and its causes, may result in actions that have to be carried out to improve the situation. This often requires a time investment before benefits can be experienced. An additional measurement, a second follow-up, could provide more insights into the development of job demands over time.

In sum, the results of the quantitative analyses suggest that the intervention was related to positive improvements in job demands and job satisfaction. In addition, results indicate that satisfaction with the communication about the intervention was related to improvements in autonomy and job satisfaction. Furthermore, results show that an increased dialogue between employees and management was related to less of a decrease in job demands.

The interviews provided more detailed information about the success of the implementation process. These results showed that working groups have put effort in the communication about the work stress prevention approach towards employees. However, the working group members and the intervention facilitator mentioned that it was challenging to keep employees involved and they believed that improvements could be made in relation to communication. Considering the results of the quantitative analyses, it is worthwhile to invest in good communication. For future interventions it is recommended to plan more meetings with all personnel to inform and involve them also during the implementation phase (Step 4), since the focus group (Step 2) and brainstorm sessions (Step 3) with all personnel were highly appreciated by employees.

Results from the interviews suggest that the intervention has increased the dialogue on stress between employees, as well as between employees and management and raised the attention for managing work stress. Participants explicitly mentioned the dialogue within the school on work stress as a key value of the work stress prevention approach. However, the effects of the increased dialogue between employees and management are somewhat inconclusive considering the fact that an increased dialogue between employees and management was related to less of a decrease in job demands.

### Strengths and weaknesses

A strength of the present study is that it involved the evaluation of an intervention implemented at five different schools, each with its own organizational context, making it possible to draw more general conclusions about the work stress prevention approach as such. In addition, the mixed method design that was used, combining quantitative data based on questionnaires and qualitative data based on interviews, and the inclusion of implementation factors in the quantitative analyses, make it possible to get a more detailed insight into the implementation process and the results of the intervention as experienced by employees. Although the sample sizes of the different schools were too small to make a comparison between schools, the response rates at the schools were quite high (at pretest as well as posttest response rates were higher than 70%) and group analyses could be performed using multilevel techniques. It has to be noted that, like the schools that participated in this study, primary schools in the Netherlands are quite small in relation to other countries (e.g. US), which may have consequences for the generalizability of the results.

The absence of a control group makes it a bit more difficult to attribute changes between baseline and follow up to the intervention. Implementation factors were measured to get an indication of the success of the implementation and were added in the analyses to explain changes between baseline and follow up on the job demands, resources and outcomes. Although the results suggest that satisfaction with communication about the intervention, an important indicator for the implementation success, is related to intervention effects on autonomy and job satisfaction, additional research is needed to look further into the mechanisms of different implementation factors (e.g. participation, involvement, communication, dialog).

For future research it would be interesting to look again at effects of the intervention and at the influence of the implementation factors. Adding a third measurement might give more insight in the effects in time and the sustainability of the effects.

## Conclusion

Despite the limitations that are discussed above, the study shows a decrease of job demands and an increase in job satisfaction in the schools that implemented the stress prevention approach. The study has provided valuable insights into the impact of the implementation of the work stress prevention approach linking the level of implementation of the intervention to improvements in autonomy and job satisfaction. Results of the study underline the importance of communication about the intervention as part of the implementation process, impacting the effectiveness of the intervention on autonomy and job satisfaction.

## Data Availability

Due to the privacy of the participants, the dataset generated and analysed during the study is not publicly available. On reasonable request data are available from the corresponding author.

## Abbreviations

PRIMA-EF: European framework for psychosocial risk management
H1–4: Hypothesis 1–4
WMO: Medical Research Involving Human Subjects Act
JCQ: Job content questionnaire
UWES: Utrecht work engagement scale
ICC: Intraclass correlation coefficient
